# Automated diagnosis of myositis from muscle ultrasound: Exploring the use of machine learning and deep learning methods

**DOI:** 10.1371/journal.pone.0184059

**Published:** 2017-08-30

**Authors:** Philippe Burlina, Seth Billings, Neil Joshi, Jemima Albayda

**Affiliations:** 1 Applied Physics Laboratory, Johns Hopkins University, Laurel, Maryland, United States of America; 2 Division of Rheumatology, Johns Hopkins School of Medicine, Baltimore, Maryland, United States of America; National Institutes of Health, UNITED STATES

## Abstract

**Objective:**

To evaluate the use of ultrasound coupled with machine learning (ML) and deep learning (DL) techniques for automated or semi-automated classification of myositis.

**Methods:**

Eighty subjects comprised of 19 with inclusion body myositis (IBM), 14 with polymyositis (PM), 14 with dermatomyositis (DM), and 33 normal (N) subjects were included in this study, where 3214 muscle ultrasound images of 7 muscles (observed bilaterally) were acquired. We considered three problems of classification including (A) normal vs. affected (DM, PM, IBM); (B) normal vs. IBM patients; and (C) IBM vs. other types of myositis (DM or PM). We studied the use of an automated DL method using deep convolutional neural networks (DL-DCNNs) for diagnostic classification and compared it with a semi-automated conventional ML method based on random forests (ML-RF) and “engineered” features. We used the known clinical diagnosis as the gold standard for evaluating performance of muscle classification.

**Results:**

The performance of the DL-DCNN method resulted in accuracies ± standard deviation of 76.2% ± 3.1% for problem (A), 86.6% ± 2.4% for (B) and 74.8% ± 3.9% for (C), while the ML-RF method led to accuracies of 72.3% ± 3.3% for problem (A), 84.3% ± 2.3% for (B) and 68.9% ± 2.5% for (C).

**Conclusions:**

This study demonstrates the application of machine learning methods for automatically or semi-automatically classifying inflammatory muscle disease using muscle ultrasound. Compared to the conventional random forest machine learning method used here, which has the drawback of requiring manual delineation of muscle/fat boundaries, DCNN-based classification by and large improved the accuracies in all classification problems while providing a fully automated approach to classification.

## Introduction

Imaging plays an important role in the assessment of muscle diseases, providing additional information to the clinician about the presence, severity, extent and activity of the disease [1, 2]. Although MRI has been considered the gold standard imaging modality for myopathies, it can be expensive, time-consuming, and difficult to obtain in patients with implants and pacemakers.

In recent years, the use of muscle ultrasound has become an important evaluation tool in neuromuscular diseases given its ease of use, lack of contraindications, and improved resolution for soft tissue structures [3–5]. In myopathies like muscular dystrophies, where increased connective tissue and fatty replacement is well visualized as increased echogenicity [6–8], quantitative assessments of echointensity have been found to correlate with functional status and worsening disease [9, 10]. Ultrasound however, can be subject to issues of operator and interpreter bias, and given dependence on echointensity changes, there is difficulty comparing results across different systems, hampering its widespread use. Various methods including quantitative ultrasound, or backscatter analysis [11, 12] have been employed to overcome some of these problems.

Our study focuses on myositis, an immune-mediated inflammatory muscle disease. Dermatomyositis (DM) and polymyositis (PM) are treatment responsive diseases, affecting primarily proximal muscles, with skin involvement in DM. Inclusion body myositis (IBM) preferentially affects the quadriceps and distal limb muscles and is refractory to standard treatment leading to severe muscle atrophy and fat replacement. Muscle and soft tissue changes in myositis can take the form of edema within and around muscles, fatty infiltration, subcutaneous reticulation and calcification [13]. Detecting and quantifying pathology as seen on ultrasound in the various stages and types of diseases is a challenging problem for this group, particularly for those changes which can reverse with treatment. Muscle inflammation and edema in the important active stage of disease do not seem to be discriminated well by a simple assessment of muscle echointensity. An early study using muscle ultrasound in myositis showed lower echointensities with increased muscle thickness in acute myositis [14]. Other studies in juvenile dermatomyositis however, have found that acutely, muscle echointensity first increases then normalizes with successful therapy [15]. The chronic stage of myositis where there is fatty replacement and fibrosis is easily discernible however, with higher echointensities and decreased muscle thickness [14]. Studies in IBM show good discrimination for the disease when screening affected muscles like the flexor digitorum profundus or gastrocnemius [16, 17].

We hypothesized that given the varying types of pathologies involved in myositis, and the different structures affected, extraction of multiple features or whole image analysis may be more ideal for the task of myositis evaluation. For example in edema, there may be a loss of perimysial echoes and a “see through” effect where underlying bone is still noted to be distinct despite increase in echointensity [3]. In dermatomyositis, there can be thickening of the fascia, subcutaneous inflammation, and patchy muscle involvement [18, 19]. These types of changes may only be appreciated by considering the entire image and not the muscle alone.

In this study, we investigate the use of computer-aided diagnostics (CAD), taking into account the entire image, which can make the muscle assessments more reproducible and accurate [20, 21]. Computer algorithms can leverage, detect and quantify image biomarkers and features which an operator may not always be able to do in a consistent fashion. The emergence of novel machine-learning techniques, including deep learning, may therefore have relevance for computer aided myopathy diagnostics.

A simplified taxonomy of terms in that domain and used subsequently in this paper is as follows: artificial intelligence (AI) is the broad field of computer science concerned with designing systems capable of intelligent reasoning and interacting with the environment. Machine learning (ML) is a subfield of AI, with goals of developing algorithms that can perform predictions on data. This generally works by building a model from training data (e.g., statistical model) which then allows one to perform inference on new data, for example by doing classification [22] or regression [23]. Deep learning (DL) [24, 25] is a subfield of ML which makes use of neural networks consisting of a multi-layered cascade of mathematical functions through which input data is processed to infer class labels [24, 25]. The mathematical functions performed by the network involve millions of parameters that are automatically learned using training data with known class labels. One technique in deep learning commonly used on image data is deep convolutional neural networks (DCNN), which help reduce the vast number of network parameters by convolving the input image with small reusable filters.

Conventional machine learning methods broadly follow a common design pattern [26, 27]: first a set of image features that fit the problem are hand selected and computed, then these features are pooled together to form feature vectors that are used to train and test a classifier such as Support Vector Machines (SVM) [22, 28] or Random Forests (RF) [29, 30]. These conventional approaches to engineered feature design may result in a set of features that are poorly chosen or too specialized to a given training dataset, which can lead to suboptimal performance and poor generalization. In general, conventional approaches may rely too much on the skill and craft of the algorithmic designer at selecting these features.

On the other hand, deep learning methods such as DCNNs [24, 25, 31], produce features that are not designed or selected by an engineer. For DCNNs in particular, image features are learned automatically from the data. Additionally, DCNNs implement all stages of a processing pipeline including feature computation, combination and final classification, all in an end-to-end model. While DCNN methods also trace their roots back many decades, recent technological and algorithmic advances have led to dramatic performance improvements for general purpose image classification. It is now possible to achieve certain tasks (e.g., whole image classification) with accuracy on par with humans. The factors mentioned above have motivated the use of DCNNs for the automated muscle disease diagnostic classification task in this study.

In sum, we explore the use of machine learning in ultrasound-based myositis assessment, particularly the use of deep learning techniques versus more conventional methods for muscle classification. As a starting point, the goal of this pilot study is to determine whether these techniques can detect changes in normal muscle versus those affected by myositis and then distinguish between types of myositis among those affected.

## Materials and methods

We first describe the data acquisition used in this study. This data has been made publicly available at https://github.com/jalbayd1/myopathy_US

### Standard protocol approvals, registrations, and patient consents

This study was approved by the Johns Hopkins University School of Medicine Institutional Review Board. All subjects were over the age of 18 and signed informed consent prior to study procedures.

### Subjects

Normal (N) subjects were recruited from the university staff and outside volunteers. Normal controls were required to have no neuromuscular or neurological disease, display normal strength and be in otherwise good health. Patients with polymyositis (PM), dermatomyositis (DM) and inclusion body myositis (IBM) were recruited from the Johns Hopkins Myositis Clinic in Baltimore, Maryland (Table 1). Patients were classified as dermatomyositis if they met Bohan and Peter criteria for definite or probable dermatomyositis [32, 33], or dermatomyositis by muscle biopsy using European Neuromuscular centre (ENMC) criteria [34]. Patients were classified as inclusion body myositis if they met 2011 ENMC criteria for clinicopathologic or clinically defined IBM [35]. Patients were classified as polymyositis if they met Bohan and Peter criteria for polymyositis with a compatible muscle biopsy, or carried a myositis specific or associated antibody and were not DM or IBM.

**Table 1:** Types of pathologies considered.

Abbreviation	Pathology/Disease	Notes
N	Normal	Control, no muscle disease
PM	Polymyositis	Muscle inflammation, proximal muscle involvement
DM	Dermatomyositis	Muscle and skin inflammation, proximal muscle involvement
IBM	Inclusion Body Myositis	Treatment refractory, with distal muscle involvement

Normal subjects were screened by questionnaire and strength testing. For patients with myositis, the creatine phosphokinase (CPK) level closest to the time of ultrasound evaluation was recorded, along with duration of symptoms of weakness (in months). All subjects underwent muscle strength testing, using the Medical Research Council scale which was then transformed to a modified Kendall’s 0-10 scale [36] and averaged per individual. Myositis specific and associated antibodies were recorded when present.

### Ultrasound acquisition protocol and tissue delineation

**Fig 1:**
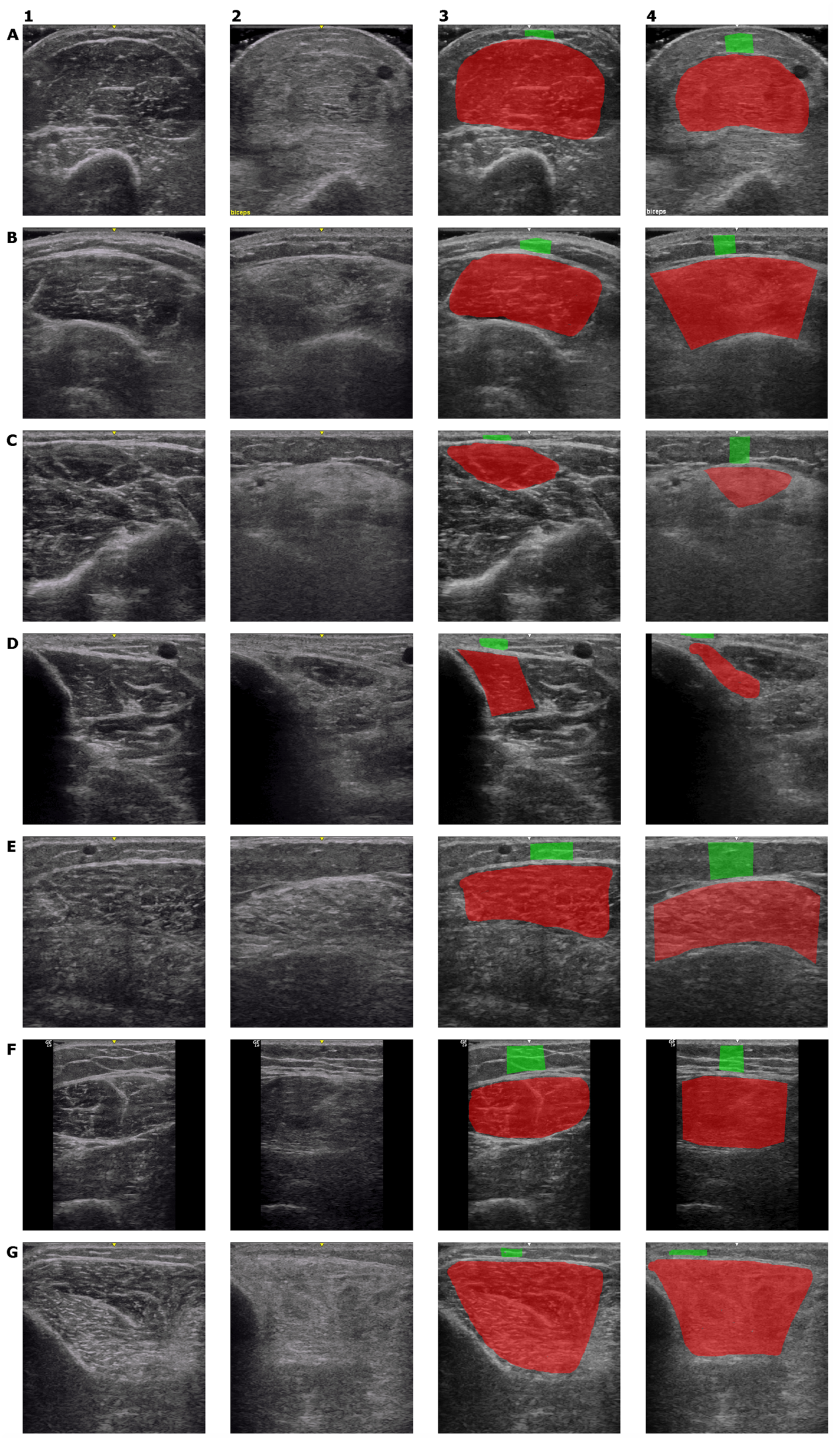
Example ultrasound images. Examples of ultrasound images for both healthy and affected individuals are shown for each muscle group studied. Each row represents one muscle group. The first column contains images of healthy individuals, whereas the second column contains images of patients suffering from myositis. The third and fourth columns show the manual segmentations of muscle and fat tissues corresponding to these images as red (for muscle) and green (for subcutaneous fat) overlays. The muscle group/disease type represented by each row are as follows. A: biceps/DM. B: deltoid/PM. C: FCR/IBM. D: FDP/IBM. E: gastrocnemius/PM. F: rectus femoris/PM. G: tibialis anterior/IBM.

Ultrasound images were acquired using a GE Logiq E system (GE, Fairfield, CT, USA) outfitted with a 12 MHz linear array transducer. Imaging parameters remained constant throughout the study with frequency at 10 Mhz, gain at 40, and dynamic range at 87 with cross beam and other enhancers turned off. Seven muscle groups were imaged bilaterally per subject (deltoids, biceps, flexor carpi radialis, flexor digitorum profundus, rectus femoris, tibialis anterior and gastrocnemius). Depth was set at 4 cm for all muscles except the rectus femoris, which was set at 6 cm. The focal zones (four focal points) were distributed evenly along the depth of the image. In this protocol, we controlled for position on the muscle, with a maximum of three B-mode images independently acquired of the transverse (cross sectional) view of each muscle to account for changes in echointensity with slight positional changes of the probe. For a few patients, only two views were captured for some muscles. In the end, this resulted in a dataset of 3214 muscle images captured for our experiments. The resulting input images had pixel resolutions (width × height) of 476 × 499 and 318 × 499 when imaging at depths of 4 cm and 6 cm, respectively. Examples of ultrasound images for healthy and diseased individuals are shown in Fig 1 for each muscle group.

### Gold-standard annotation

Gold-standard assignment of disease type was performed for each muscle and was assigned by the clinical expert (JA) based on known clinical diagnosis.

### Taxonomy of 2-class classification problems studied

The study considered three separate binary (two-class) automated muscle ultrasound diagnostic classification problems (Table 2). These address the following questions. First, whether imaged muscles can be differentiated as healthy or affected by myositis. In particular, problem (A) looks at this question with the entire cohort (N versus IBM, PM, DM), while problem (B) uses only healthy individuals and IBM (N versus IBM), which is the most severe type of myositis given lack of treatment-response. Finally, we consider problem (C), which looks at only those individuals with myositis: We focus our classification on Inclusion Body Myositis compared to other myositis (PM, DM versus IBM) as IBM has a different type of muscle involvement from the other two and is clinically treated differently.

**Table 2:** Problems studied.

Problem	Cohort inclusion	Clinical problem	Number of patients	Number of images
A	All subjects	2-class patient diagnostics N vs. {IBM, PM, DM}	80	3214
B	Normal + IBM only	2-class patient diagnostics N vs. IBM	52	2107
C	Myopathic patients only	2-class diagnostics {PM, DM} vs IBM	47	1901

Problem A involves mixing all types of recruited patients (normal and any type of myositis). We are interested in distinguishing normal muscle from diseased muscle (N versus PM, DM, IBM). In Problem B, we seek to differentiate out the extremes of the spectrum on imaging, Normal from Inclusion Body Myositis (N versus IBM). Problem C involves only affected individuals. We attempt to differentiate IBM which has a different type of muscle involvement, from PM and DM (PM, DM versus IBM)

### Classification via deep learning and deep convolutional neural networks (DL-DCNN)

We used a DCNN [24, 37–39] for automated muscle classification, a deep learning approach capable of solving complex and generic image classification and medical image analysis tasks [24, 40–43]. A DCNN can be thought of as a processing network consisting of numerous layers including convolutional (e.g., filtering/matching layers), activation and pooling layers. A simple interpretation of DCNNs is that they compute image features at different levels of abstraction, using convolutional filters whose weights are obtained directly from the data by using training via a backpropagation process. Backpropagation learns the filter weights that result in the best fitting function, mapping the DCNN input (training muscle images) to the output diagnostic labels. DCNNs combine the computed features and output a final probability score characterizing whether the muscle image belongs to a specific diagnosis class (e.g., healthy vs. diseased).

**Fig 2:**
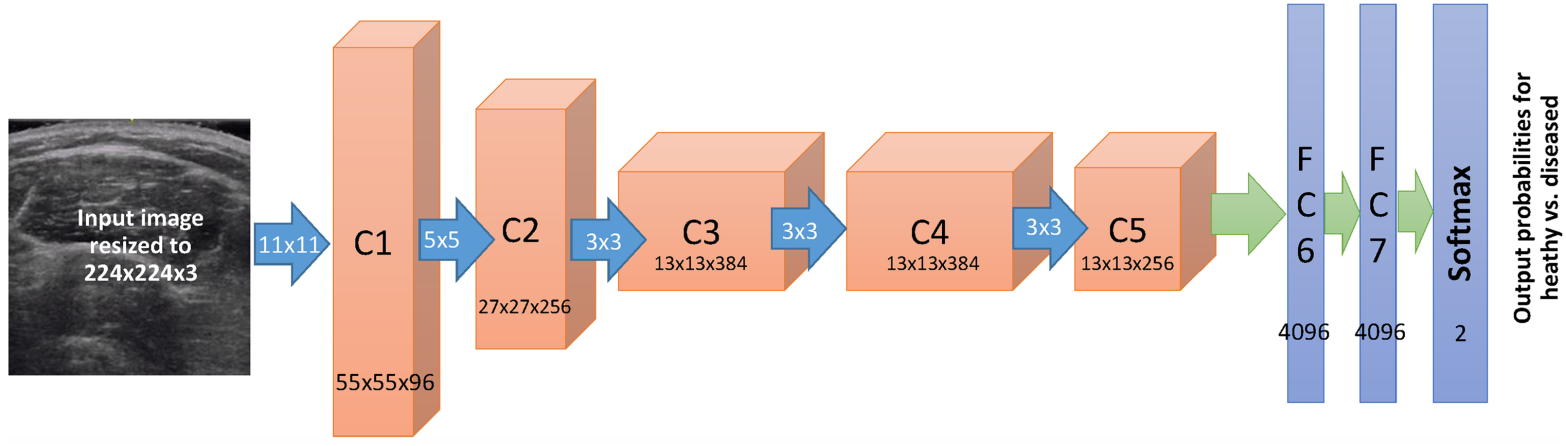
DCNN architecture. This figure depicts the architecture of the AlexNet DCNN used in this study. The muscle images are input at left and the final class probabilities for categorization are output at right. Layers C1-C5 are convolutional layers, followed by fully connected layers (FC6 and FC7), and finally by the Softmax layer outputting the probabilities of the image corresponding to each disease. (For further architectural details, see the original AlexNet paper by Krizhevsky [44]).

We trained a specific DCNN for each of the problems (A), (B) and (C). We used the Keras framework with Theano as back end and the AlexNet network model [44] (Fig 2). For weight initialization we used AlexNet weights pre-trained on the ImageNet dataset [45] consisting of over 1 million images and one thousand classes. We then replaced the (Softmax) last layer of the network to output a two class probability for the diagnosis and retrained all the network weights for all layers of the network by using labeled muscle ultrasound training images. Training was done using stochastic gradient descent with a learning rate = 0.001, a momentum = 0.9, and Nesterov momentum enabled, which was used to minimize a categorical cross-entropy loss function. Using a step decay learning rate scheduler, the learning rate was decreased with multiplicative *γ* = 0.1 every 100 epochs. Training termination used an early stopping method, which monitored the validation loss, and stopped training after 100 epochs of no improvement.

### Classification via conventional machine learning using random forests (ML-RF)

We compared the DCNN-based automatic diagnosis to a more conventional machine learning method consisting of first computing image features and then automatically classifying the disease using a random forest (RF) classifier. To be useful, these low-level image features must be computed within delineated regions of the image corresponding to muscle and fat tissues. ITK-Snap [46] (Kitware, Clifton Park, NY, USA) was used by the study physician (JA) to manually segment the desired muscle and subcutaneous fat tissues from each US image. Examples of these segmentations are shown in Fig 1. Since it required manual image delineation by a clinician the method is therefore semi-automated.

#### Image features

We included as image features the absolute and relative measures of echointensity for muscle and fat, as these were shown to be useful biomarkers for ultrasound image-based myopathy diagnosis [6, 47]. We used five echointensity features including mean and standard deviation of echointensity of muscle as well as fat, and the ratio of these means. The computed ultrasound image features were augmented with Nakagami and Haralick image features. Nakagami is a probability distribution well suited for modeling US echointensity [48]. Its two parameters describing shape and scale (*m*, *ω*) can characterize scattering conditions and tissue microstructure. Lastly, we also combined the above features with 13 Haralick image features, which also characterize image texture and the underlying tissue structure [21, 49]. All together, these features formed a 22-element image feature vector fed to a random forest classifier.

#### Random forests

Random forests (RF) [29] were used to perform classification on each image feature vector. A random forest is a collection of random decision trees, where at each node of the tree a randomly selected subset of features is chosen to make a decision. Training of the tree is done using a random subset of the training data. This leads to a collection (forest) of decision trees each of which forms a unique classifier. The collection of trees then takes as input the computed image features vector and each tree provides a vote to automatically classify the disease.

#### Data analysis

For each problem (P), and each method—either the deep learning or random forest approach—we assessed performance using the following metrics: accuracy, equal to 100% minus the classification error rate; sensitivity, equivalent to true positive rate or recall, which measures the proportion of positive examples that are correctly classified as positive; specificity, the true negative rate, which measures the proportion of negative examples that are correctly classified as negative; positive predictive value (PPV) and negative predictive value (NPV), which are the proportions of positive and negative predictions that are actually true positive and true negative; Cohen Kappa score, which discounts agreement occurring due to chance; and finally, the positive and negative likelihood ratios (LR+ and LR-, respectively), which help determine whether a test result is useful in changing the prior probability that a condition exists. For each metric, the standard deviation of the values across different folds (explained next) was calculated and provides a measure of the confidence interval (COI) (as under a Gaussian assumption the 95% COI is about twice the standard deviation).

Performance was measured using a conventional N-fold cross-estimation of the above metrics. We split the data randomly into *N* = 5 subsets (folds); for each of five runs, the folds were rotated between four folds used for training and one fold used for testing. A separate classifier was trained for each run, for each classification method (either DL-DCNN or ML-RF), and for each of the problems (A)–(C). For each problem and classification method an average accuracy and a standard deviation were then calculated across folds. When sub-dividing the data into different folds, care was taken to ensure that all images of the same muscle—which are considered unique entities—were always assigned to the same fold.

For the DCNN method, an additional hold out data subset was carved out as validation data; for each N-fold run, 70% of the total data was used for training, 10% for validation, and 20% for testing. The hold-out validation data (independent from the testing data) was used to decide when to stop training the DCNN based on the validation loss.

## Results

### Demographics

We recruited 80 subjects (49 female and 31 male) including 33 normal, 19 IBM, 14 PM, and 14 DM. Subject ages ranged from 23 to 84 years of age; ages distributed by decade included 5 subjects at 20-29 years of age, 10 at 30–39 years, 11 at 40–49 years, 17 at 50–59 years, 20 at 60–69 years, 13 at 70–79 years, and 4 at 80–89 years. Patients with IBM had a much longer duration of disease (as measured from onset of weakness) than the PM and DM groups. The IBM group had moderate elevations of muscle enzyme levels (CPK) and were the weakest group overall. Muscle enzyme levels were highest for the PM group which was made up mostly of immune-mediated necrotizing myopathies. These patients were already on treatment and had largely preserved strength despite muscle enzyme elevations. The DM group on the other hand had the lowest CPK levels but displayed more weakness than the PM group likely due to multiple factors including muscle atrophy, hypomyopathic forms and more multi-system disease. Antibody specificities are reported in Table 3. For patients with IBM, no myositis specific antibodies were found, but the cytosolic 5'-nucleotidase 1A (c5n1A) antibody was not routinely tested for.

**Table 3:** Demographics and subject characteristics table: Mean and standard deviation (parenthesized) are provided. Duration of weakness is expressed in units of months. N/A indicates that duration of weakness and CPK was not collected for normal subjects. For the associated antibodies rubric, the parenthesized values indicate the number of patients falling in the category. Also the abbreviations are as described next. C5N1A: cytosolic 5'-nucleotidase 1A; SRP: signal recognition particle; HMGCR: 3-hydroxy-3-methyl-glutaryl-CoA reductase; TIF1gamma: transcriptional intermediary factor 1 gamma.

	IBM	PM	DM	Normal
**Number of Subjects**	19	14	14	33
**Male / Female**	10 / 9	2 / 12	5 / 9	14 / 19
**Age**	64.0 (10.2)	59.4 (14.5)	52.6 (17.1)	50.9 (15.5)
**Duration of weakness**	134.8 (91.8)	63.1 (68.8)	57.2 (38.3)	N/A
**CPK (24-195)**	566 (596)	1547 (1808)	242 (292)	N/A
**Strength**	8.5 (2.1)	9.4 (1.6)	8.9 (2.3)	10 (0)
**Associated Antibodies**	c5N1a not routinely tested	HMGCR (6), RNP (2), Ku (2), PL-12 (2), mitochondrial (1), SRP (1)	TIF1-*γ* (3), SAE (2), PL-7 (1), Jo-1 (5), PM-Scl (1), EJ (1), Mi-2 (1)	N/A

### Classification performance assessment and N-fold cross estimation of accuracy

Classification performance was assessed by using the metrics described earlier in the data analysis subsection and computed for each of the problems (A)–(C) defined in Table 4.

**Table 4:** Classification performance and standard deviation (parenthesized) for each problem.

P	Method	Accuracy	Sensitivity	Specificity	PPV	NPV	Kappa	LR+	LR-
A	DL-DCNN	76.2 (3.1)	81.6 (3.6)	68.6 (6.1)	79.1 (3.3)	72.1 (4.1)	0.51 (0.07)	2.69 (0.66)	0.27 (0.05)
	ML-RF	72.3 (3.3)	77.3 (1.8)	65.0 (6.8)	76.3 (3.8)	66.4 (3.1)	0.42 (0.07)	2.29 (0.56)	0.35 (0.05)
B	DL-DCNN	86.6 (2.4)	81.2 (6.0)	89.9 (2.6)	83.0 (3.5)	89.0 (3.0)	0.71 (0.05)	8.43 (2.19)	0.21 (0.07)
	ML-RF	84.3 (2.3)	71.8 (4.5)	91.9 (2.2)	84.3 (3.8)	84.4 (2.1)	0.66 (0.05)	9.35 (2.54)	0.31 (0.05)
C	DL-DCNN	74.8 (3.9)	66.6 (4.7)	80.7 (5.8)	71.6 (6.1)	77.1 (2.7)	0.48 (0.08)	3.71 (1.19)	0.42 (0.06)
	ML-RF	68.9 (2.5)	59.2 (2.1)	75.9 (4.2)	63.9 (4.0)	72.1 (1.4)	0.35 (0.05)	2.51 (0.42)	0.54 (0.04)

The resulting performance metrics and standard deviation for the three classification problems (A)-(C) and each method (DL-DCNN and ML-RF) are reported in Table 4. In this table it can be seen, in particular, that performance ranged from a high of 86.6% accuracy for the best performing method (DL-DCNN) on classification problem (B), to a low of 68.9% for the worst performing method (ML-RF) on problem (C). From the table one can see that that the single metrics performance (accuracy, Kappa score, LR+ and LR-) gave a preference for the DL-DCNN approach when compared to the ML-RF method.

It should be noted that a higher LR+ (and conversely a lower LR-) indicate better performance. With regard to LR+ and LR- values in the results table, we can note that these do make a difference with regard to altering the probability of the condition being present before and after the test was performed. This is especially the case for problem B where both methods can be said to yield a moderate to large increase in post test probability of having the disease should the test come up positive, with a preference for the DL-DCNN method. The DL-DCNN method also has a good influence on the post-test probability for problems A and C with regard to positive values and negative values when compared to ML-RF. The same can be said on the influence on the post probability should the test come up negative.

## Discussion

In past studies, parameters such as muscle echointensity, relative echointensity of muscle compared to subcutaneous fat, as well as texture characteristics were shown to be useful for neuromuscular diseases [50–52]. These were also used in our conventional ML-RF method. In this study, compared to ML-RF, deep-learning-based classification by and large improved accuracy in all problems. This is interesting and supports the notion that features that are manually selected—while effective—are probably suboptimal or may not be exhaustive for full disease characterization when compared to data-driven features found via deep learning. We surmise that other aspects not captured by these selected features are somehow computed by the deep learning approach. The enhanced performance results of the DL-DCNN approach is also complemented by other distinct advantages. As opposed to the ML-RF approach used in this study, which required the clinician to perform manual muscle delineation to yield usable features (semi-automated), the DL-DCNN approach is applied to the entire image, and is also fully automated which would have implications for simpler clinician workflows.

The overall performance results obtained in this small study are encouraging. The best results were obtained for problem B, which involves marked differences with regard to muscle condition and image presentation—i.e., normal muscles versus IBM, where atrophy and highly echogenic muscle predominate. By contrast, Problem A, which considers the problem of normals versus all myositis in general—yields images with many more variations of presentation for the computer to disambiguate in terms of differences in pathology, which likely explains lower performance. Indeed, our sample of patients with PM, DM, and IBM span a range of acute to chronic cases, mild to moderate disease, with patients already on treatment (except for IBM). This is actually more challenging for the algorithm given the variety of pathology and the inclusion of near-normal appearing muscles that are instead annotated as diseased cases. This fact can negatively influence the computer aided assessment by providing training exemplars of images that seem normal but belong to cases annotated as affected. We would expect that obtaining more homogeneous patient groups would significantly improve performance. Performance was lowest for distinguishing treatable (PM, DM) versus treatment refractory (IBM) disease. This is not surprising given the fact that it involves only myositis variants and has the smallest cohort of patients and images to train from, including nearly half of the images and patients when comparing problem (C) to problem (B)

Of note, this study used all muscle data irrespective of muscle type for machine inference of the disease type. This also is a challenging problem in that the machine would have to learn to recognize together both the muscle type and the pathology case. A simpler method—also likely to yield more accurate results—would consist of posing the diagnostic inference problem for each muscle separately: this would essentially result in “informing” the algorithm of the muscle type and would likely lead to better performance given that each muscle looks different, and that subgroups of myositis affect certain muscles preferentially. We hope to pursue this type of analysis differentiating between muscle types (as was done for example in a study of myositis patients, which concentrated only on biceps brachii [21]) as well as grouping all the muscles together per each individual in the future. This however would also require more patient data and was a limitation of our study.

Given the rarity of this disease and the difficulties with recruitment, our population represents a real world convenience sample, and the small numbers represent a significant limitation. Another limitation was that patients and controls were not age and sex matched. It has been shown that muscle echointensity does increase slightly with age [53, 54], and diseased muscle usually shows much higher echointensities than expected for age [14]. Though not matched, we did include healthy controls ranging from the age of 23 to 74 which would also allow for assessment of other age-related changes in parameters. Disease duration could also not be controlled as patients with IBM present with slowly progressive weakness leading to later detection, versus DM and PM which come to the attention of a physician earlier due to symptoms. Another point of contrast for the groups is that effective treatment in DM and PM can improve weakness as well as the quality of the muscle as seen on ultrasound, and this is not true of IBM where changes continue to accrue. Therefore, rigorous matching to control for differences in the patient groups could not be done for this population. However, given inherent differences in the nature of these diseases which are actually capitalized on clinically, we feel that results shown are still valid.

For this study, clinical exam, strength testing, antibody results, and histopathologic criteria were used to clinically categorize patients into disease subgroups and ensure the exclusion of IBM from the PM group. This served as the gold standard adjudication of disease categories. Further correlation with these parameters was beyond the scope of this study but is of interest for future investigation. The incorporation of additional clinical information such as muscle strength, muscle enzyme levels, treatment and the like, along with image data to perform machine inference, may potentially enhance classification performance [55] and could mimic a clinician’s process. The addition of this ‘side channel’ information in DCNNs is a potential avenue for future improvement of the myositis diagnostic algorithm, particularly since distinguishing between types of disease is usually difficult when considering the result of only imaging [56]

Recognizing the limitations of our study, we hope to accrue more patients in each disease subgroup, including enough with both early and late disease to allow for more granular analyses, as well as be able to separate out those with inactive disease (and near-normal appearing muscles), from those with clinical activity. We also plan to compare ultrasound findings with MRI, and when available histopathology, to understand the nature of the changes detected (edema, fat replacement, etc). With improvement in classification performance, we hope to test these methods on a different cohort, particularly using a different ultrasound system.

While this study sought to classify images into disease subtypes in very challenging conditions, other aspects of the diagnostic problem may constitute good candidate applications for the use of machine learning methods: these include for example looking at longitudinal follow-up in known disease, or classifying acute versus chronic muscle changes (picking up edema).

Summing up all considerations, and taking into account the aforementioned challenges, the results of this study, while preliminary and exploratory, provide an instructive foray into the use of deep learning methods for muscle disease classification. We demonstrate the potential of combining machine learning and deep learning methods in particular, with muscle ultrasound, for myositis assessment. To our knowledge, this is the first application of deep learning to muscle imaging. Therefore, one of the values of this pilot study is in laying a foundation and providing a baseline performance assessment for future work in this arena.

## Conclusion

This study considers the development of machine learning methods for automatically or semi-automatically classifying inflammatory muscle disease, in particular myositis. We show that when compared to the conventional machine learning method that requires careful clinician delineation, the deep learning approach used here always performs better, while being fully automated and requiring no user intervention.
